# Fast-evolving microRNAs are highly expressed in the early embryo of *Drosophila virilis*

**DOI:** 10.1261/rna.041657.113

**Published:** 2014-03

**Authors:** Maria Ninova, Matthew Ronshaugen, Sam Griffiths-Jones

**Affiliations:** Faculty of Life Sciences, University of Manchester, Manchester, M13 9PT, United Kingdom

**Keywords:** microRNAs, *Drosophila*, development, evolution, embryogenesis

## Abstract

MicroRNAs are short non-protein-coding RNAs that regulate gene expression at the post-transcriptional level and are essential for the embryonic development of multicellular animals. Comparative genome-scale analyses have revealed that metazoan evolution is accompanied by the continuous acquisition of novel microRNA genes. This suggests that novel microRNAs may promote innovation and diversity in development. We determined the evolutionary origins of extant *Drosophila* microRNAs and estimated the sequence divergence between the 130 orthologous microRNAs in *Drosophila melanogaster* and *Drosophila virilis*, separated by 63 million years of evolution. We then generated small RNA sequencing data sets covering *D. virilis* development and explored the relationship between microRNA conservation and expression in a developmental context. We find that late embryonic, larval, and adult stages are dominated by conserved microRNAs. This pattern, however, does not hold for the early embryo, where rapidly evolving microRNAs are uniquely present at high levels in both species. The group of fast-evolving microRNAs that are highly expressed in the early embryo belong to two *Drosophilid* lineage-specific clusters: mir-310∼313 and mir-309∼6. These clusters have particularly complex evolutionary histories of duplication, gain, and loss. Our analyses suggest that the early embryo is a more permissive environment for microRNA changes and innovations. Fast-evolving microRNAs, therefore, have the opportunity to become preferentially integrated in early developmental processes, and may impact the evolution of development. The relationship between microRNA conservation and expression throughout the development of *Drosophila* differs from that previously observed for protein-coding genes.

## Abstract (precis)

In this paper, the authors have used deep sequencing and state-of-the-art computational analysis tools to address the relationships of microRNA evolutionary origins, rates, and expression patterns in a developmental context in two species, *Drosophila melanogaster* and *Drosophila virilis*. They find that early stages of embryogenesis are uniquely characterized by high levels of rapidly evolving and lineage-specific microRNAs. They propose that fast-evolving and newly emerged microRNAs may be implicated in the evolution of insect development. Their analyses suggest that the early embryo is a more permissive environment for microRNA changes and innovations.

## INTRODUCTION

MicroRNAs are short, non-protein-coding RNAs that regulate the production of protein from coding mRNAs by sequence-specific translational inhibition or transcript degradation. Since the discovery of lin-4 and let-7 as important regulators of developmental timing in *Caenorhabditis elegans* (Lee et al. 1993; Reinhart et al. 2000), increasing evidence has highlighted the crucial role of microRNAs in diverse developmental processes including cell fate decision, differentiation, axis formation, morphogenesis, and organogenesis in both protostome and deuterostome animals (Kloosterman and Plasterk 2006). Conservative computational predictions suggest that microRNAs target one- to two-thirds of protein-coding genes in animals (Grün et al. 2005; Lewis et al. 2005; Friedman et al. 2009).

For protein-coding genes, it is well-established that there is a strong positive correlation between sequence conservation and expression levels (Subramanian and Kumar 2004), and similar trends have also been also reported for microRNAs (Liang and Li 2009; Shen et al. 2011; Meunier et al. 2012; Roux et al. 2012). The functional relevance of nonconserved microRNAs is the subject of much debate, as they are often reported to be expressed at low levels or during restricted stages of development (Berezikov 2006; Chen and Rajewsky 2007; Lu 2008; Liang and Li 2009; Meunier et al. 2012; Roux et al. 2012). Despite a general positive correlation between sequence conservation and expression, conservation of the protein-coding transcriptome is not uniform across different stages of development (Davis et al. 2005; Hazkani-Covo et al. 2005; Cruickshank and Wade 2008; Roux and Robinson-Rechavi 2008; Domazet-Lošo and Tautz 2010; Kalinka et al. 2010; Irie and Kuratani 2011; Kalinka and Tomancak 2012; Quint et al. 2012). For example, the protein-coding transcriptome of adult animals has been shown to be less conserved than that of the embryo in both invertebrates and vertebrates (Domazet-Lošo and Tautz 2010). This is suggested to be a consequence of significant canalization of particular cellular processes, deployed at specific developmental stages, which are less robust to the consequences of mutations (Davis et al. 2005; Hazkani-Covo et al. 2005; Cruickshank and Wade 2008; Roux and Robinson-Rechavi 2008; Domazet-Lošo and Tautz 2010; Kalinka et al. 2010; Irie and Kuratani 2011; Kalinka and Tomancak 2012; Quint et al. 2012). It has been suggested that microRNAs have birth and death dynamics distinct from protein-coding genes, and that they act as buffers contributing to canalization of developmental processes (Hornstein and Shomron 2006; Peterson et al. 2009). In the light of these observations, we explored the relationship between microRNA evolutionary rates and their expression levels in a developmental context.

Arthropods have been widely used as models to explore the intersection between development and evolution (Angelini and Kaufman 2005; Peel et al. 2005; Peel 2008). Most of our current understanding of microRNA evolution and function in arthropods comes from studies in *Drosophila melanogaster* and related Drosophilid species (Ruby et al. 2007). As a leading model organism, the microRNA repertoire of *D. melanogaster* has been well-characterized experimentally. Some *D. melanogaster* microRNAs are members of widely conserved animal microRNA families; however, a substantial fraction of its microRNA complement is lineage- or species-specific. For example, fruit flies (*Diptera*) and beetles (*Coleoptera*) diverged ∼300 mya (Wiegmann et al. 2009) and share only one-third of their microRNA gene set, with the remaining microRNAs emerging after their split (Marco et al. 2010).

The majority of fruit fly microRNAs are expressed in distinct and characteristic spatio-temporal patterns throughout development, as evident from in situ hybridization, Northern blotting, and next-generation sequencing (Aravin et al. 2003; Aboobaker et al. 2005; Biemar et al. 2005; Ruby et al. 2007). However, existing staged embryonic data sets to date are limited to a single species, *D. melanogaster*, and sample relatively broad developmental time points. In order to obtain a more detailed picture of microRNA dynamics during arthropod embryogenesis, and simultaneously provide a basis for comparative studies, we generated precisely timed small RNA libraries of developing and adult *Drosophila virilis*. *D. melanogaster* and *D. virilis* diverged ∼63 million years ago (Tamura et al. 2004) and represent the two main subgenera of fruit flies: *Sophophora* and *Drosophila*. We used state-of-the-art RNA similarity search tools to identify and explore the evolutionary histories of orthologous microRNAs between *D. melanogaster* and *D. virilis* and calculate the rates of evolutionary divergence. We then assessed the relative expression levels of microRNAs evolving at different rates across developmental time in *D. virilis*. We find that early embryogenesis is uniquely characterized by elevated levels of fast-evolving microRNAs. Although with lower temporal resolution, previous data in *D. melanogaster* shows a similar trend, suggesting this phenomenon is conserved. We propose that this pattern reflects either specific functional roles for fast-evolving microRNAs in early development or that there is selection against mutations in microRNAs that are expressed in later stages due to pleiotropic consequences. Detailed examination of these fast-evolving early development microRNAs shows that they are members of two clusters that emerged in the last common ancestor of the Drosophilids.

## RESULTS

### Identification of origins and evolutionary rates of *D. virilis* microRNAs

**FIGURE 1.**
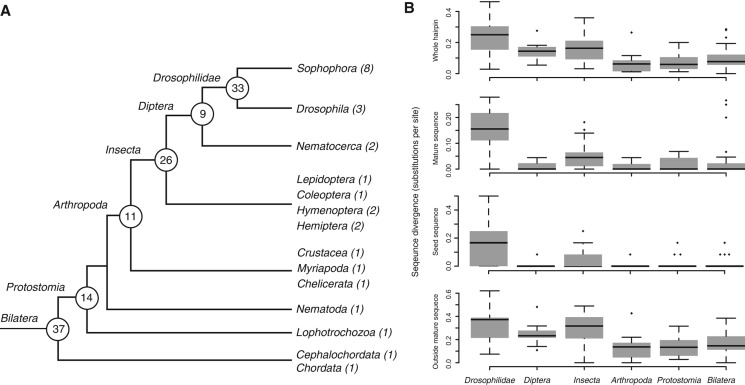
Origins and divergence of *Drosophila* microRNAs. (*A*) Tree shows taxa of the animals in which homologs of the *Drosophila* microRNAs were searched. MicroRNAs were assigned an evolutionary age depending on the most distant species in which a member of the same family was identified. The number of microRNAs that emerged on each branch is shown. (*B*) Numbers of substitutions per site between *D. melanogaster* and *D. virilis* orthologous microRNAs of different evolutionary origins, in the whole hairpin, the mature sequence, the seed sequence, and the stem extension and loop region (outside mature). *X*-axis labels indicate the age of each microRNA, as in *A*.

We used the experimentally supported set of *D. melanogaster* microRNA sequences to search for conserved microRNAs in *D. virilis*. In total, we identified 131 *D. virilis* homologs, 130 of which have an unambiguous 1-to-1 ortholog in *D. melanogaster* (as determined by best reciprocal hits and synteny). In order to gain an insight into the relationship between microRNA age and rate of sequence divergence, we estimated the evolutionary origins of the microRNA families that are conserved between *D. virilis* and *D. melanogaster*. MicroRNA origins were inferred using the parsimony method, based on the range of species in which homologous sequences could be confidently identified (see Materials and Methods). The number of conserved Drosophilid microRNAs that emerged on each phylogenetic branch is shown in Figure 1A (see Supplemental Table S1 for the full list of queried species). Previous studies in *D. melanogaster* suggested that the microRNAs that emerged before the diversification of the Drosophilids evolve slower than those that appeared afterward (Nozawa et al. 2010). We aligned the *D. virilis* and *D. melanogaster* orthologs and calculated the substitutions per site for microRNAs from the different evolutionary age groups in the whole hairpin region, the mature sequences, the 6-mer seed region (positions 2–7), and in the loop and stem extension (Fig. 1B). Consistent with previous studies (Lu et al. 2008; Nozawa et al. 2010) and on a broader time scale, we find that the more ancient microRNAs have undergone fewer changes between *D. melanogaster* and *D. virilis* than microRNAs that have emerged more recently in all parts of the hairpin, including the functional mature sequences and seed regions. Thus, the sequences of young microRNAs change faster than old microRNAs. The few outliers—microRNAs to which an old age was assigned but which have high evolutionary rates—are homologs of ancient microRNAs that emerged more recently by duplication. There is a significant positive correlation between the evolutionary rates of the hairpin sequence inside and outside the mature microRNA region (Pearson's *r* = 0.38; *P* < 10^−5^).

### MicroRNA developmental expression profile in *D. virilis*

In order to create a detailed data set of microRNA expression across *D. virilis* development, we generated small RNA libraries from embryos at precisely timed 2-h intervals covering the first 16 h of its embryogenesis, 16- to 30-h embryos representing later stages until hatching, third instar larvae, and adult animals. Samples were sequenced on the Illumina HiSeq 2000 platform yielding ∼20 million reads for each sample. Eighty to ninety-six per cent of these reads mapped to the *D. virilis* genome with no more than one mismatch. The small RNA content was dominated by reads of ∼30 nt, followed by reads of ∼22- to 23-nt length (Supplemental Fig. S1A). This distribution is expected, because the 30-nt *Drosophila* 2S ribosomal RNA is abundant in small RNA libraries. Between 44% and 90% of the short reads (19–24 nt) mapping to the genome in each library map to microRNAs (Supplemental Fig. S1B). We found evidence of expression for 117 of the 131 computationally predicted homologs of *D. melanogaster* microRNAs. In addition, using a conservative pipeline for microRNA annotation previously established in our lab (Marco et al. 2010), we identified 13 candidate novel microRNAs unique to the *virilis-repleta* group (Supplemental Fig. S2). The majority of the novel *D. virilis-*specific microRNAs are localized in introns of annotated genes and/or are clustered in the genome, supporting the hypothesis that new microRNAs emerge more frequently in regions that are actively transcribed (Berezikov et al. 2010; Campo-Paysaa et al. 2011; Marco et al. 2013b). Numbers of reads mapping to *D. virilis* microRNAs in each library are shown in Supplemental Table S2. Two microRNAs, mir-286 and mir-956, have read counts that are one to two orders of magnitude higher than the next most highly expressed microRNA in the corresponding library; these sequences were excluded from further analyses.

**FIGURE 2.**
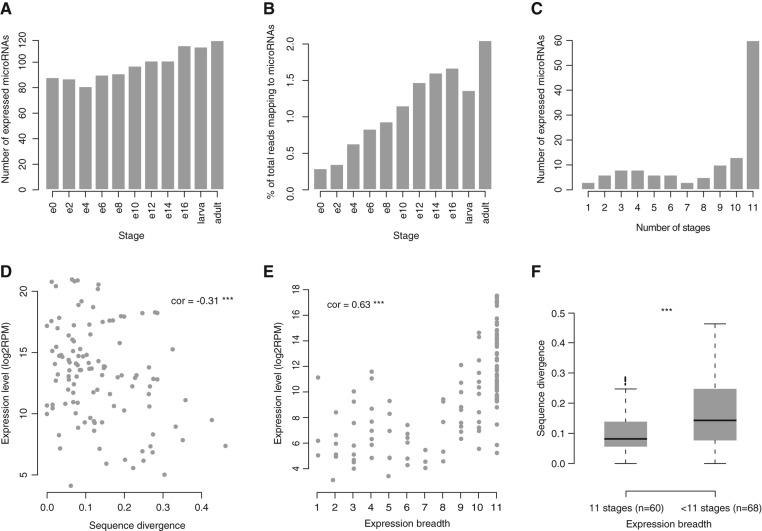
MicroRNA expression in *D. virilis* small RNA libraries. Samples include nine embryonic stages: 0- to 2-h embryo (e0), 2- to 4-h embryo (e2), etc., 16- to 30-h embryo (e16), larvae and adults. (*A*) Number of individual microRNAs detected in each library. (*B*) Percentage of reads mapping to microRNAs with respect to the total number of reads mapping to the genome in each library. (*C*) MicroRNA expression breadth. Graph shows the numbers of microRNAs expressed in a number of developmental stages from 1 (single stage) to 11 (all stages). (*D*) Scatter plot of the relationship of summed microRNA expression level in all libraries and microRNA hairpin sequence divergence. (*E*) Scatter plot of the relationship of summed microRNA expression levels and microRNA expression breadth. Pearson's product-moment correlation value is shown in the body of *D* and *E*. (***) *P* < 0.001. (*F*) Box plot of microRNA hairpin sequence divergence of microRNAs expressed at all developmental stages (11 stages) and microRNAs expressed at fewer developmental stages (<11 stages). Mean values are significantly different [Student's *t*-test; (***) *P* < 0.001].

Both the absolute numbers of expressed microRNAs and the total proportion of reads that map to microRNAs increase over developmental time (Fig. 2A,B). Approximately half of the microRNAs were detected in all examined libraries, while the rest have narrower temporal expression profiles (Fig. 2C). We find a small yet significant negative correlation between the sum of microRNA expression levels across all libraries and sequence divergence (between *D. virilis* and *D. melanogaster*) of the hairpin sequence (Fig. 2D) and a positive correlation between the expression level and the temporal breadth of expression (Fig. 2E). Figure 2E shows the expression level as the average across libraries where a given microRNA is expressed. A similar correlation is seen if expression level is represented either by the sum or the maximum expression level across all tissues (data not shown). MicroRNAs expressed in all sampled time points (11 developmental stages) have significantly fewer substitutions in their hairpin structure than those absent from one or more libraries (Fig. 2F), and the correlation between microRNA temporal expression range and sequence divergence is negative (ρ = −0.35, *P* < 10^−5^). Altogether, our results suggest that conserved microRNAs are more highly and more broadly expressed in *D. virilis* than more rapidly evolving microRNAs. This is consistent with data for both microRNAs and protein-coding genes from other organisms (Subramanian and Kumar 2004; Shen et al. 2011).

### Abundance and conservation of microRNAs at different stages of *D. virilis* development

**FIGURE 3.**
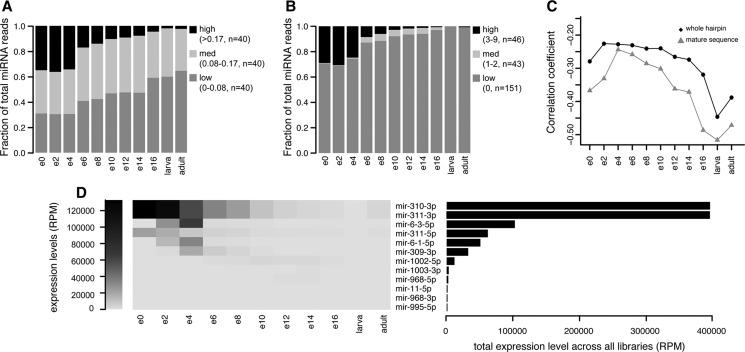
Expression patterns of microRNAs of different evolutionary rates throughout the development of *D. virilis*. Stages are labeled as follows: 0- to 2-h embryo (e0), 2- to 4-h embryo (e2), etc., 16- to 30-h embryo (e16), larvae, and adults. (*A*,*B*) MicroRNAs hairpins (*A*) and mature microRNA sequences (*B*) were divided into three groups depending on the substitution rates between *D. melanogaster* and *D. virilis.* Bars represent the relative expression levels of the microRNAs from each group (with low substitution rates, medium substitution rates, and high substitution rates) at each stage of development. The number of microRNAs and the sequence divergence range for each bin is shown in the legend. Note that different arms of the same hairpin are treated separately in *B*. (*C*) Correlation of microRNA evolutionary rates at the level of the whole hairpin and the mature sequences and microRNA expression levels throughout development. Vertical axes represent Spearman's correlation coefficient (ρ) obtained for each stage; values are statistically significant (*P* < 0.05). (*D*) Expression levels and patterns of individual mature microRNAs with three or more substitutions in their sequence between *D. melanogaster* and *D. virilis*. The heat map on the *left* shows the expression levels of the microRNA from this group throughout development, and the bar graph on the *right* reflects the total expression level of each microRNA in all libraries. MicroRNAs are sorted by their total expression levels across all libraries, and microRNAs with negligible total expression levels of below 1000 RPM are not shown.

Despite the overall correlation between expression levels and evolutionary conservation of protein-coding genes (Subramanian and Kumar 2004), transcriptome studies in a developmental context have indicated that the evolutionary age and both sequence and expression divergence of protein-coding transcripts varies between different stages of embryogenesis in both animals and plants (Davis et al. 2005; Domazet-Lošo and Tautz 2010; Kalinka et al. 2010; Quint et al. 2012). This motivated us to explore the relationship between microRNA expression levels and evolutionary rates at each individual stage of fruit fly development. We grouped *D. virilis* microRNAs according to their substitution rates, measured across the microRNA hairpin, into three equally sized bins—microRNAs with low, medium, and high numbers of substitutions per site. Figure 3A shows the relative contribution of microRNAs from each group to the overall microRNA landscape at each developmental stage. In the early embryo, the relative levels of the microRNAs from the three groups are roughly similar. However, as the developmental time progresses, the levels of fast-evolving microRNAs decrease, reaching a minimum in the larva and in the adult organism, where conserved microRNAs dominate. We next asked if this trend holds for the functional products of the microRNA hairpin—the mature arms. It is well-established that the majority of substitutions in the microRNA hairpins are outside the mature sequences, as shown in Figure 1B (Nozawa et al. 2010; Shen et al. 2011). Indeed, most mature products from the orthologous microRNAs between *D. virilis* and *D. melanogaster* are perfectly conserved. Nevertheless, 43 mature sequences have undergone one or two substitutions since the two species diverged, and 46 have three or more differences. Figure 3B shows the relative levels of mature sequences from these groups at different developmental stages. Again, we observe elevated levels of fast-evolving microRNAs in the early embryo compared with later stages. This pattern holds not only using values relative to the total number of reads mapping to microRNAs but also when microRNA read counts are normalized against the total number of reads mapping to the genome (Supplemental Fig. S3).

We further estimated the relationship between microRNA sequence divergence and expression levels across the developmental time of *D. virilis* using the Spearman's rank correlation test. This measure is independent of read count normalization and avoids biases introduced by a small number of microRNAs expressed at levels orders of magnitude higher than the median. The resulting correlation coefficients at each developmental stage are shown in Figure 3C. In larvae, adult animals, and the latest embryonic stages, sequence divergence and expression level exhibit a moderate negative correlation. In contrast, the correlation between microRNA sequence divergence and expression levels in the early embryo is weaker, consistent with the observations that expression levels of poorly conserved microRNAs are higher at this stage.

Analysis of pre-existing data sets of microRNA expression during different *D. melanogaster* developmental stages showed patterns very similar to those observed in *D. virilis* (Supplemental Fig. S4; see Supplemental Table S3 for *D. melanogaster* data set descriptions and GEO accession numbers), suggesting that early expression of fast-evolving microRNAs is a conserved phenomenon within the Drosophilid lineage. We also assessed our *D. virilis* and *D. melanogaster* microRNA data by computing the transcriptome age and divergence index (TAI and TDI)—an approach previously used to estimate the sequence divergence of plant and animal transcriptomes across development (Domazet-Lošo and Tautz 2010; Quint et al. 2012). The results strongly support the trends observed using correlation analysis, with the early embryo exhibiting the youngest and most rapidly evolving microRNA profile (Supplemental Fig. S5).

We inspected the levels and the temporal expression patterns of the most divergent mature microRNAs (with 3–9 substitutions). Most rapidly evolving microRNAs (34 of the 46) are expressed at low levels (below 1000 RPM, or less than 1/1000 of the total reads mapping to microRNAs). Figure 3D shows the temporal expression patterns of the remaining fast-evolving mature microRNAs whose total expression levels exceed 1000 RPM. The observed patterns in the early embryo are largely due to a small number of microRNAs that are present at high levels in the initial stages of development and not expressed at later stages. We do not see any fast-evolving microRNAs present at high levels in the late embryo or at any other developmental stage. This overall expression pattern is highly conserved in *D. melanogaster* (Supplemental Fig. S4D).

The highly expressed, rapidly evolving microRNAs in the early embryo are organized in the genome in two large clusters: mir-310∼313 cluster and mir-309∼6 cluster. These clusters have complex evolutionary histories, discussed below, and although some of their members are related to deeply conserved microRNAs, the clusters themselves appear to be innovations in the Drosophilid lineage.

### Evolution and expression of the mir-310∼313 cluster

**FIGURE 4.**
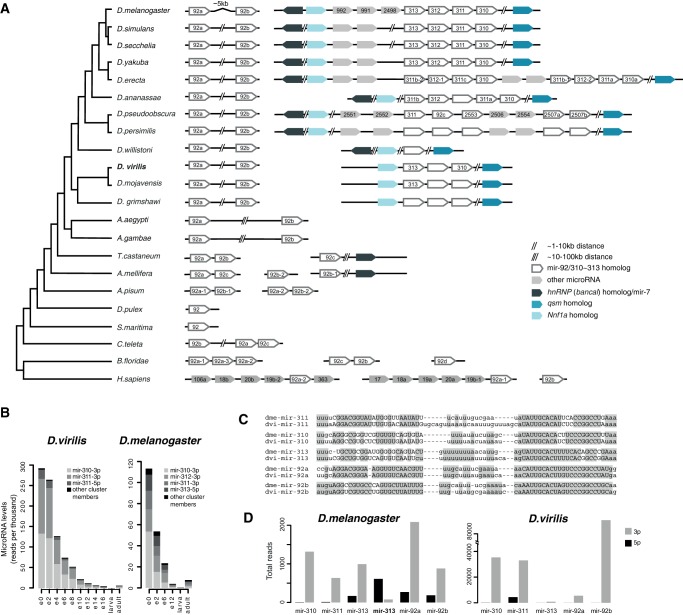
Evolution and expression of the mir-310∼313 cluster members. (*A*) Schematic diagram of the evolutionary history of the extended mir-92/mir-310 family. Individual chromosomes or scaffolds and presented with a black line and oriented in the direction of the corresponding microRNA transcript(s). MicroRNAs annotated in miRBase are labeled, while homologs found by BLAST/INFERNAL searches are blank. Homologs of flanking protein-coding genes are shown as colored blocks. (*B*) Expression levels of the members of the mir-310∼313 cluster in small RNA libraries from different developmental stages of *D. melanogaster* and *D. virilis*. Horizontal axis labels are as in Figures 2 and 3. (*C*) Alignments of mir-310 family members in *D. melanogaster* and *D. virilis*. Mature arms are marked in uppercase and conserved sites between 1-to-1 orthologs are highlighted. (*D*) Total number of reads across experiments mapping to the 5′ and the 3′ arms of mir-310∼313 cluster members in *D. melanogaster* and *D. virilis*.

We have used synteny information to reveal a detailed picture of the evolutionary relationships of the microRNAs of the mir-310∼313 cluster (Fig. 4A). The cluster consists of several homologous microRNAs in close proximity to one another. In *D. melanogaster*, it includes four coexpressed members: mir-310, mir-311, mir-312, and mir-313, which appear to be expressed independently of the neighboring mir-2498, mir-991, and mir-992 sequences (Ruby et al. 2007; Ryazansky et al. 2011). In the *Obscura* group, the cluster is heavily rearranged and harbors additional nonhomologous microRNAs, while in the *Drosophila* subgenus, we find three clustered homologs of the mir-310 family. In all fruit flies, the cluster is localized in an intergenic region between the protein-coding genes *qsm* and *Nnf1a* and *bl* (which, in turn, harbors the ancient mir-7). The mir-310∼313 family members were shown to have emerged in fruit flies by duplication of an ancient mir-92 family member (Lu et al. 2008), and our analysis supports this. A separate locus harbors two members of the mir-92 family, and its organization is also highly conserved in all flies. Clusters of two mir-92 homologs are also found in other insects, but their genomic context differs. Interestingly, mir-92 homologs in *Tribolium castaneum* (tca-mir-92c) and *Apis mellifera* (ame-mir-92b-1) are localized near the *bl*/mir-7 homolog, suggesting deeper origins of the Drosophilid mir-310∼313 cluster than previously thought. Although members of the mir-310∼313 cluster belong to the evolutionarily ancient mir-92 family, taking duplication events into consideration, the mir-310∼313 cluster itself is obviously younger, with most of its members unique to the fruit fly lineage.

Mature products of members of the mir-310∼313 cluster, particularly miR-310-3p and miR-311-3p in both species, and dvi-miR-311-5p, dme-miR-312-3p, and dme-miR-313-5p, are present at very high levels in the earliest stages of *D. virilis* and *D. melanogaster* development, including the 0- to 2-h time point which comprises predominantly prezygotic embryos and thus maternally loaded RNAs (Fig. 4B). In fact, these microRNAs constitute more than a quarter of the total microRNA content at that stage, declining over developmental time, with only trace amounts found in adult samples. Previous studies of microRNA expression patterns in the *D. melanogaster* embryo did not detect nascent transcripts for the mir-310∼313 cluster in the fruit fly embryo (Aboobaker et al. 2005) by in situ hybridization, and we also could not identify mir-310∼313 transcription using a nascent transcript in situ approach in *D. virilis* early stages. These data suggest that microRNAs from the mir-310∼313 cluster are maternally deposited rather than expressed in the embryo in the two species. Despite this conserved accumulation via maternal loading, members of the mir-310∼313 cluster show unusually high rates of evolution (Lu et al. 2008). Figure 4C shows that there are multiple substitutions in the predicted homologs between the two species not only in the microRNA stem, loop, and the 5′ arms but also in the 3′ arms from which most mature products are generated (Fig. 4D). Furthermore, while all members of the *D. virilis* and three members of the *D. melanogaster* mir-310∼313 cluster express most of their mature products from the 3′ arm, dme-mir-313 generates a higher level of mature products from its 5′ arm (Fig. 4D). Therefore, the mir-310∼313 cluster is rapidly evolving in three aspects—divergence of mature microRNA sequences, microRNA arm usage, and local duplications/losses of microRNA genes.

### The evolution and expression of the mir-309∼6 cluster

**FIGURE 5.**
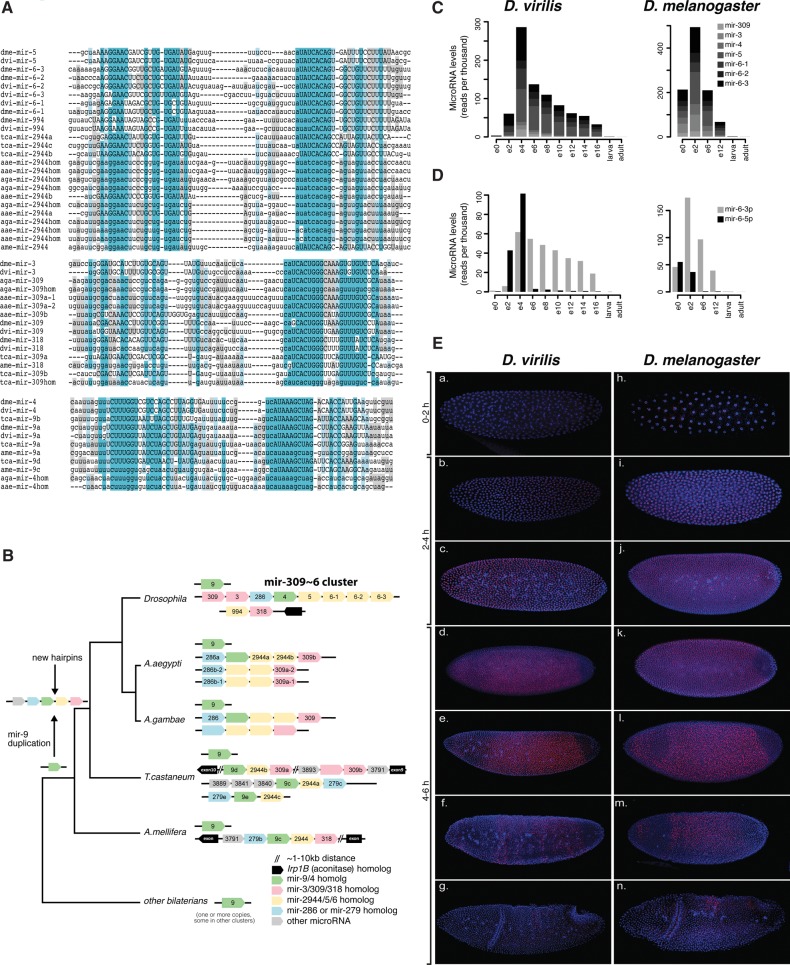
Expression and evolution of the mir-309∼6 cluster. (*A*) MicroRNA sequence alignments for mir-309/3/318, mir-5/6/2944, and mir-9/mir-4 homologs in *D. melanogaster, D. virilis, Anopheles gambiae, Aaedes aegypti, Tribolium castaneum*, and *A. mellifera*. Known mature sequences are shown in uppercase, and conserved sites are highlighted. (hom) Denotes homologs that are discovered in our study but not annotated in miRBase. (*B*) Evolutionary history of the mir-309∼6 cluster. The organization and content of microRNA clusters containing homologs of members of the mir-309∼6 in Drosophilids are shown for *D. melanogaster, D. virilis, A. gambiae, A. aegypti, T. castaneum*, and *A. mellifera*. Black lines represent different chromosomes/scaffolds. MicroRNAs annotated in miRBase are labeled, and homologs are color-coded but blank. Note that some species have one or more additional paralogs of mir-9, which are not shown, for simplicity. (*C*) Expression levels of mature mir-309∼6 cluster products throughout the development of *D. virilis* and *D. melanogaster.* (*D*) Expression levels of the 5′ and 3′ arms of *D.virilis* and *D. melanogaster* mir-6 paralogs throughout the development of the two species. (*E*) Expression of mir-309∼6 primary transcript in embryos from *D. virilis* and *D. melanogaster*. All embryos are oriented with ventral to the *top* and anterior to the *right*. The mir-6 primary transcript is expressed ubiquitously in precellular embryos of *D. virilis* [(a) 0–2 h; (b,c) 2–4 h] and *D. melanogaster* (h–j), except for the pole bud cells. In stage 5 embryos of both species (d,e,k,l,), the expression is repressed first from the posterior, and next in the anterior region. During the next stages (f,g,m,n), repression continues, until a final dorsal stripe disappears by the time of gastrulation.

The mir-309∼6 cluster encodes a polycistronic transcript producing eight microRNAs: mir-309, mir-3, mir-286, mir-4, mir-5, mir-6-1, mir-6-2, and mir-6-3. Its content, organization, and localization are highly conserved in all 12 fruit flies with sequenced genomes. Members of the mir-309∼6 cluster have previously been described as Drosophilid-specific. We manually curated multiple sequence alignments of all cluster members and used the state-of-the-art INFERNAL tool and synteny to predict homologs (Fig. 5A,B). We find that members of the mir-309∼6 cluster are of different evolutionary ages. The mir-4 sequence has the most ancient homolog, mir-9, which is conserved in all bilaterian organisms. Other members of the mir-9 family produce dominant mature microRNAs from their 5′ arm, whereas mir-4 generates dominant mature products from the 3′ arm. mir-286 and mir-279 homologs in *Tribolium* and *Apis* have sequence similarity in their 3′ arm, yet computational methods do not identify them as related. Thus, this microRNA is either young or highly diverged between the different insect lineages. mir-3 family members (mir-309 and mir-3 in Drosophilids, and mir-309a/b in *Tribolium*) are related to the mir-318 family, which is conserved between fly and honeybee; mir-5, mir-6, and mir-994 also appear to be related to each other, as well as to the mir-2944 family members found in other insects (Fig. 5A), but mir-5 and mir-6 mature products originate from different arms. Again, sequence alignments indicate that these homologs have diverged significantly in the different insect lineages.

The mir-994∼318 cluster in Drosophilids, mir-9d∼309b in *Tribolium*, and mir-9c∼318 in *Apis* are all localized in antisense orientations near or within a predicted intron of a gene for *aconitase*. This, together with the microRNA ordering, suggests that these clusters are likely to be orthologous. Based on the data shown in Figure 5, A and B, we suggest that the mir-4 family resulted from a duplication and arm-switching of mir-9 in the last common ancestor of insects, in a process similar to that previously described for mir-10 and mir-993 (Griffiths-Jones et al. 2011). The founding members of the mir-309/3/318, mir-5/6/2944, and mir-286 families then emerged by de novo hairpin formation near mir-4 and expanded by tandem duplications. Recent data from honeybee (Zondag et al. 2012) showed that mir-2944 is present at high level in the early embryo, suggesting that an early microRNA transcript harboring mir-4, mir-5/6/2944, and mir-309/3/318 was present in the last common ancestor of flies and bees. The microRNAs in this cluster have then undergone various duplications, rearrangements, and individual gains and losses during insect evolution. In the fruit flies, one copy of the cluster was rearranged and expanded, resulting in the mir-309∼6 cluster. Thus, although the individual members of the cluster have homologs in other insects, its content and organization is unique to Drosophilids. Because of multiple duplication and loss events, it is difficult to infer the exact time of emergence of all eight microRNAs in the cluster. For example, there are three mir-6 paralogs, of which at least two appear to have emerged after flies and mosquitoes split. As with all other duplicated microRNAs, although individual members of the cluster may belong to ancient microRNA families, some of the extant copies are likely to be significantly younger.

Mature microRNA sequences from members of the mir-309∼6 cluster are most highly expressed during *D. virilis* gastrulation (4–6 h) and in the most biologically similar data set of *D. melanogaster* (2–6 h), accounting for 30%–50% of all microRNA reads in these stages (Fig. 5C). The most diverged mature microRNAs (with three or more substitutions) that are highly expressed are miR-309-3p and the 5′ arms of mir-6-1 and mir-6-3. Interestingly, during the narrow period of cluster expression (e2, e4), both miR-6-3p and miR-6-5p products are present at high levels (Fig. 5D). After that, the more diverged miR-6-5p rapidly decreases, while miR-6-3p sequences remain long after, suggesting differential regulation of the arm stability. Previous studies showed that in *D. melanogaster*, the mir-309∼6 primary transcript is expressed ubiquitously in the precellular embryos (Biemar et al. 2005), under the control of the zinc-finger early *Drosophila* activator (Zelda) (Liang et al. 2008). Transcription then becomes restricted from the ventral and posterior regions and is finally abolished by the end of gastrulation (Biemar et al. 2005). Comparison of the regions upstream of the mir-309∼6 cluster in *D. melanogaster* and *D. virilis* shows high sequence similarity, including conserved binding sites for Zelda and other TAGteam members in close proximity to the transcriptional start site (Supplemental Fig. S6). Consistent with this, in situ hybridization shows that the spatio-temporal pattern of mir-309∼6 cluster expression is very similar in the two species (Fig. 5E). As an aside, we note that, although transcription occurs for a brief period of time, mature products of the cluster remain throughout the entire course of embryogenesis, highlighting the possibility that early expressed microRNAs may be able to exhibit long-term effects on later development.

## DISCUSSION

*Drosophila* microRNAs are of broad evolutionary origins and have variable rates of sequence evolution—from sequences that are perfectly conserved across the fruit fly phylogeny and have homologs across all bilaterian animals to rapidly evolving lineage-specific microRNAs. There are two technical factors that are worthy of discussion here. First, a commonly used approach for homologous microRNA identification is by sequence similarity searches using BLAST. However, some microRNAs evolve fast and escape detection by this approach. Using covariance model searches and taking into account synteny information, we uncovered numerous homology relationships that have so far escaped sequence similarity detection. We, therefore, strongly argue for the importance of these methods in the analysis of the complex evolutionary history of microRNA families and their use as markers to infer phylogeny (also discussed in Hertel et al. 2012). Second, because of their short sequence, some microRNAs diverge to the point where they cannot be confidently identified as homologs. For example, we cannot confidently determine whether mir-286 and mir-279 have evolved from a common ancestor. In that sense, microRNA evolutionary origins determined by the most distant species in which a homolog can be identified and microRNA evolutionary rates are intrinsically related. Indeed, the few outliers from the evolutionary age and sequence divergence relationship of Figure 1B—microRNAs with high divergence rates but with ancient origins—appear to have arisen by a recent duplication. This rapid divergence is likely a consequence of either relaxed selection due to redundancy and/or tight genomic linkage, in the case of clustered microRNAs (Marco et al. 2013a), or possibly adaptive evolution (Lu et al. 2008).

Our results show that the profile of microRNA expression at most stages of the fruit fly life cycle is dominated by microRNAs that have remained highly conserved within the fruit fly lineage since its split. However, detailed analyses of temporal microRNA expression patterns during discrete stages of development show that some lineage-specific and fast-evolving microRNAs are uniquely present at high levels in the early embryo. Interestingly, the only fast-evolving, highly expressed microRNAs are localized in clusters. The extant organization of these clusters in fruit flies is clearly specific to the lineage, but the presence of related loci in other taxa suggests that these hairpins may have arisen within a pre-existing microRNA harboring transcript. The unusual evolutionary dynamics of clustered microRNAs may be at least partly due to genetic linkage, as recombination between tightly linked loci is unlikely (Marco et al. 2013a).

As the microRNA sequence required for target recognition is short, each new microRNA has the potential to perturb the translation of hundreds of protein-coding mRNAs simply by chance. Previous studies have shown that gene expression divergence in the early embryo is high (Kalinka et al. 2010) and that the protein-coding transcriptome on that stage is compromised of relatively young and divergent genes (Domazet-Lošo and Tautz 2010; Quint et al. 2012). Although the explanation for this phenomenon is elusive, it is thought that early development is more robust to, or buffered from, functional consequences of change compared with later stages (Davis et al. 2005; Hazkani-Covo et al. 2005; Roux and Robinson-Rechavi 2008; Domazet-Lošo and Tautz 2010; Irie and Kuratani 2011). In the light of this hypothesis, we speculate that, due to its robustness, the early embryo is a more permissive environment for changes in existing microRNAs and acquisition of novel microRNAs.

The extended germ band stage covered by the 6- to 10-h data set in *D. melanogaster* and 6- to 12-h in *D. virilis* is often referred to as the phylotypic stage, as it represents the most homologous developmental stage between different species (Raff 1996). The phylotypic stage has recently been shown to display the oldest and most conserved protein-coding transcriptome among the entire ontogenesis of animals and plants (Davis et al. 2005; Hazkani-Covo et al. 2005; Cruickshank and Wade 2008; Domazet-Lošo and Tautz 2010; Kalinka et al. 2010; Irie and Kuratani 2011; Quint et al. 2012). In contrast, although the correlation of microRNA conservation and expression is stronger at that stage than in the earlier embryo, it is not the lowest across development. This could indicate that microRNA expression does not follow the same patterns as protein-coding transcripts. An alternative explanation follows from the fact that small RNA sequencing measures the levels of the mature product rather than the primary transcript: microRNA transcription may occur for a short period of time, while the product remains stable long after. This is analogous to the observation that transcriptome and proteome expression data are not very well-correlated (de Sousa Abreu et al. 2009; Maier et al. 2009; Schwanhäusser et al. 2011). Nonetheless, clear differences between microRNA and protein-coding gene age and expression in adult animals remain: previous studies suggest that a key characteristic of the adult transcriptome is that younger genes are more highly expressed (Domazet-Lošo and Tautz 2010), while we observe that conserved and older microRNAs have the highest levels of expression in adults.

MicroRNAs were first identified as regulators of the developmental timing in *C. elegans* (Lee et al. 1993; Reinhart et al. 2000) and have since been recognized as important players with diverse functions in animal development. Developmental functions are not restricted to deeply conserved microRNAs. For example, functional studies on the members of the mir-310∼313 and mir-309∼6 clusters in *D. melanogaster* suggest that, in the embryo, they are involved in maternal transcript turnover, morphogenesis, and apoptosis (Leaman et al. 2005; Bushati et al. 2008; Ge et al. 2011). Although the precise molecular role of these relatively young and fast-evolving microRNAs is not fully understood, their conserved expression pattern in the two major Drosophilid subgenera suggests that their expression in the early embryo represents a general property rather than a species-specific peculiarity. A recent analysis of honeybee microRNAs also reports species-specific sequences present at high levels in the early embryo (Zondag et al. 2012). MicroRNAs from the mir-430 family, which has undergone a massive expansion in fish, are also shown to be highly expressed in the early embryo of *Danio rerio* (Chen et al. 2005; Giraldez et al. 2005, 2006), and a similar pattern has been observed for mir-427 in *Xenopus* (Lund et al. 2009, 2011). In addition, relatively low correlation between microRNA evolutionary age and expression levels was observed in mouse embryonic tissues (Roux et al. 2012). The emergence of new microRNAs has been speculated to play a role in generating complexity and diversity among multicellular animals (Berezikov 2006; Sempere et al. 2006; Heimberg et al. 2008; Peterson et al. 2009; Berezikov et al. 2010; Marco et al. 2010; Griffiths-Jones et al. 2011). It is also well-established that the molecular networks that operate in early development have diversified substantially among various insect taxa (for review, see Davis and Patel 2002). Altogether, these data provide clues that early embryogenesis is particularly amenable to the emergence of novel microRNA regulation with potential evolutionary consequences.

## MATERIALS AND METHODS

### Fly culture and sample collection

Wild-type *D. virilis* were maintained on standard fly media at 25°C. For embryo collection, flies were transferred to breeding cages supplied with apple juice agar plates and supplemented with yeast paste. Plates were removed, and embryos were allowed to age for the desired periods of time under the same conditions. Embryos were then harvested on a mesh sieve, de-chorionated with 50% hypochlorite solution for ∼2 min, and thoroughly washed. An aliquot of each sample was immediately fixed and de-vitellinated as described previously (Kosman et al. 2004), and stored in methanol for staining. The remaining embryos were disrupted in 1 mL Trizol (Invitrogen), and total RNA was extracted according to the manufacturer's instructions. Wandering third instar larvae and 7- to 10-d-old adults were collected from fly culture vials and washed with 50% hypochlorite solution and distilled water, followed by Trizol total RNA extraction. The quality of the total RNA was confirmed by Agilent Bioanalyzer, and small RNA library preparation and sequencing on an Illumina HiSeq 2000 were performed at GATC Biotech, generating 50-bp reads.

### MicroRNA expression data

Adaptor sequences of the 50-bp Illumina reads from *D. virilis* libraries were removed using the Cutadapt tool (http://code.google.com/p/cutadapt/). The trimmed reads were first searched against annotated *D. virilis* rRNAs, tRNAs, snoRNAs, snRNAs, and other noncoding RNAs retrieved from FlyBase (FB2011_07) with Bowtie v0.12.7 (Langmead et al. 2009), allowing one mismatch, and reads mapping to these sequences were filtered out. The remaining reads were mapped to the *D. virilis* genome (r1.2_FB2011_07, retrieved from FlyBase), allowing one mismatch. Sequences mapping to fewer than five loci in the genome, and of lengths between 19 and 24 nt were used for detection of *D. virilis* microRNAs as described previously (Marco et al. 2010). Small RNA sequencing data sets for *D. melanogaster* were downloaded from the GEO database (Edgar et al. 2002) (see Supplemental Table S3 for accession numbers) and mapped to *D. melanogaster* microRNA hairpins retrieved from miRBase (v18) with the same parameters as for *D. virilis*. The number of reads mapping to each microRNA hairpin was used as an estimate of its expression level. Data were normalized against the total reads mapping to microRNAs in the given library, except for Figure 2B and Supplemental Figure S4, where total reads mapping to the genome were used instead. There are a few instances of paralogous microRNAs that share identical mature sequence. Because we cannot distinguish which of the loci was the source of the reads detected by deep sequencing, the total number of reads for such sequences was divided by the number of different microRNA loci to which they mapped.

### MicroRNA sequence divergence and evolutionary age

*D. melanogaster* microRNA sequences from miRBase (v18) were used as a query for sequence similarity searches using BLASTN (*w* = 4, *r* = 2, *q* = −3, *e* = 0.01) (Altschul et al. 1990) in the genomes of all sequenced members of the *Drosophila* genus, *Aaedes aegypti, Anopheles gambiae, Glossina morsitans, Bombyx mori, T. castaneum, A. mellifera, Nasonia vitripennis, Acyrthosiphon pisum, Daphnia pulex, Strigamia maritima, Ixodes scapularis, C. elegans, Capitella teleta*, and *Branchiostoma floridae* (see Supplemental Table S1 for list of genome sources and assemblies). The resulting predicted homologs were aligned with ClustalX 2.1 (Larkin et al. 2007), and alignments were inspected manually using RALEE (Griffiths-Jones 2005). The consensus curated alignments were used to build covariance models with INFERNAL v1.1 (Nawrocki et al. 2009), and we searched these models against the genomes of species in which no homologs were identified in the previous step. All hits with an E-value less than 1 were added to the existing alignments for manual inspection. Homologs of the newly discovered *D. virilis* microRNAs were identified using the same approach. MicroRNA evolutionary origins were estimated by parsimony based on the most distant species in which a homolog of a given microRNA could be identified. In the few cases of duplicated microRNAs, ages were assigned based on the oldest member of the microRNA family, even though the duplication might have occurred later in evolutionary history.

One-to-one microRNA orthologs between *D. melanogaster* and *D. virilis* were determined based on reciprocal best BLAST hits. To calculate sequence divergence of the microRNA hairpin, we used substitutions per site and Kimura's two-parameter method (Kimura 1980). The two estimates produced similar results; for simplicity, we present results with the former measure. Again for simplicity, the numbers of substitutions rather than substitutions per site for microRNA mature sequence evolutionary rates are used in Figure 3, as microRNA mature sequences are of approximately equal length (22 ± 2 nt).

MicroRNA transcriptome age and divergence indices (TAI and TDI) were computed as described previously (Domazet-Lošo and Tautz 2010; Quint et al. 2012). Here, TAI is the sum of the expression of microRNAs at a given developmental stage *s* (*e_is_*), weighted by their ages from 1 to 7 (*a_i_*, where number 1 was assigned to the youngest microRNAs conserved only in Drosophilids, and 7 to the oldest microRNAs conserved in all Bilateria) (see Fig. 1A), divided by the total counts of all microRNAs *m* expressed at that stage. As values of 7 correspond to older family ages and 1 to the youngest, high values of TAI reflect older microRNA transcriptome. TDI is calculated in a similar manner, but instead of age, the values for substitutions per site (*n*) are used. Higher TDI values, therefore, reflect expression of fast-evolving genes.

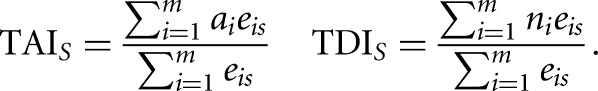

The standard deviation of these values was estimated by bootstrapping 1000 times.

### Immunohistochemistry and in situ hybridization

Approximately 1-kb fragments flanking the mir-310∼313 and mir-309∼6 clusters in *D. melanogaster* and *D. virilis* were amplified from genomic DNA of wild-type flies of the corresponding species, using the following primers: *D. virilis* mir-309∼6 cluster: 5′-TTCAGTTTTGCCAGGCTCTT-3′ (forward) and 5′-TCCCTGCGGTTAAACATAGC-3′ (reverse); *D. virilis* mir-310 cluster 5′-GCAAATCGCTGCTACAGACA-3′ (forward) and 5′-GCCCTTTCAAATGATTAACAGC-3′ (reverse); *D. melanogaster* mir-309/6 cluster: 5′-CAAAGCTTGAGGAATTTGTGC-3′ (forward) and 5′-AGTGTAAGGATCCCGCAGTG-3′ (reverse); *D. melanogaster* mir-310 cluster: 5′-CACTTGCCACTTGCAAAAGA-3′ (forward) and 5′-GCGAATTCCTTCGATTTCCT-3′ (reverse). These fragments served as templates for the synthesis of digoxigenin-labeled antisense RNA probes, and fluorescent in situ hybridization and antibody staining was performed as described previously (Kosman et al. 2004). We used sheep anti-digoxigenin primary and anti-sheep Alexa Fluor 555 secondary antibodies. Results were visualized using a Olympus FV1000 confocal microscope and processed with FiJi (Schindelin et al. 2012).

## DATA DEPOSITION

The sequencing data are deposited in the NCBI Gene Expression Omnibus, with accession no. GSE54009.

## SUPPLEMENTAL MATERIAL

Supplemental material is available for this article.
